# Zinc erythrocyte protoporphyrin as marker of malaria risk in pregnancy - a retrospective cross-sectional and longitudinal study

**DOI:** 10.1186/1475-2875-11-249

**Published:** 2012-07-30

**Authors:** Edward L Senga, Gibby Koshy, Bernard J Brabin

**Affiliations:** 1 Biochemistry Department College of Medicine, University of Malawi, Blantyre, Malawi; 2 Child and Reproductive Health Group, Liverpool School of Tropical Medicine, Pembroke Place, Liverpool, L3 5QA, UK; 3 Global Child Health Group, Emma Kinderziekenhuis, Academic Medical Centre, University of Amsterdam, Amsterdam, Netherlands

**Keywords:** Malaria, Zinc erythrocyte protoporphyrin, Pregnancy

## Abstract

**Background:**

The effects of iron interventions and host iron status on infection risk have been a recurrent clinical concern, although there has been little research on this interaction in pregnant women.

**Methods:**

Cross-sectional and longitudinal analyses were undertaken to determine the association of whole blood zinc erythrocyte protoporphyrin (ZPP) with malaria parasitaemia in pregnant women attending antenatal and delivery care at Montfort and Chikwawa Hospitals, Shire Valley, Malawi. Prevalence of antenatal, delivery and placental malaria was assessed in relation to maternal ZPP levels. The main outcome measures were prevalence of peripheral and placental *Plasmodium falciparum* parasitaemia and odds ratios of malaria risk.

**Results:**

A total of 4,103 women were evaluated at first antenatal visit, of whom at delivery 1327 were screened for peripheral and 1285 for placental parasitaemia. Risk of malaria at delivery (peripheral or placental) was higher in primigravidae (p < 0.001), and lower (peripheral) with use of intermittent preventive anti-malarials during pregnancy (p < 0.001). HIV infection was associated with increased malaria parasitaemia (p < 0.02, peripheral or placental). Parasitaemia prevalence was lower in women with normal ZPP levels compared to those with raised concentrations at both first antenatal visit (all gravidae, p = 0.048, and at delivery (all gravidae, p < 0.001; primigravidae, p = 0.056). Between first antenatal visit and delivery women who transitioned from raised (at first antenatal visit) to normal ZPP values (at delivery) had lower peripheral parasitaemia prevalence at delivery compared to those who maintained normal ZPP values at both these visits (all gravidae: 0.70, 95%CI 0.4-1.1; primigravidae: 0.3, 0.1-0.8). In regression analysis this difference was lost with inclusion of HIV infection in the model.

**Conclusions:**

Raised ZPP concentrations in pregnancy were positively associated with *P. falciparum* parasitaemia and were probably secondary to malaria inflammation, rather than indicating an increased malaria risk with iron deficiency. It was not possible from ZPP measurements alone to determine whether iron deficiency or repletion alters malaria susceptibility in pregnancy.

## Background

The effects of iron interventions and host iron status on infection risk have been a recurrent clinical concern [1] and this interaction has been examined in several meta-analyses [2-4] and a Cochrane Review [5]. Concerns have focused mainly on possible harmful effects of treating iron deficiency [5], rather than on the effects of iron status itself on infection risk. There has been little research on this interaction in pregnant women, in whom iron supplementation studies have focused on anaemia and birth-weight outcomes rather than infection [6].

Two previous cross-sectional studies in Malawi [7] and Tanzania [8] reported that maternal iron deficiency was associated with reduced risk of placental malaria. Iron status was assessed using either the serum transferrin receptor: log ferritin ratio [7], or serum ferritin corrected for elevated C - reactive protein [8]. These studies suggested that iron biomarkers may be useful predictors of malaria risk in pregnancy. An alternative iron biomarker, red cell zinc protoporhyrin/haem (ZPP), has the relative advantage of low cost and simplicity. Although in children with infection, inflammation, or high malaria parasite densities, ZPP specificity may be reduced [9-11]. In a cohort study in young children, baseline ZPP levels alone were used as an iron biomarker for defining iron deficiency and for assessing malaria incidence over 600 days [12]. This study showed that anaemic children with iron deficiency who received iron and folic acid supplementation had lower malaria-related adverse events than children receiving placebo, although the analysis did not control for malaria treatments given. No studies were identified which examined host iron status based on ZPP as a predictor of malaria risk in pregnant women. The rationale for the present study was to determine the association of ZPP with malaria risk in pregnancy. Cross-sectional malaria prevalence was assessed at first antenatal visit, and also in longitudinal ZPP categories for women seen at both first antenatal visit and at delivery, allowing a possible directional influence of gestational iron status to be assessed.

The data for the analysis was collected between 1992–1995 in a large study, which described the pattern of malaria and anaemia during pregnancy and associations with pregnancy outcomes [13-15]. The analysis of this large data set allowed the utility of ZPP to be assessed as a potential biomarker of malaria risk both at first antenatal visit, prior to commencement of routine haematinic supplementation with iron and folic acid, and at delivery following several weeks of haematinic supplementation. This is the first analysis of malaria risk in pregnancy using ZPP as a biomarker of host iron status.

## Methods

### Study design

Cross-sectional and longitudinal analyses of whole blood zinc erythrocyte protoporphyrin (ZPP) were conducted in order to determine its association with malaria parasitaemia prevalence in pregnant women attending for antenatal and delivery care.

### Study location

Study enrolment was between March 1993 and July 1994 in Chikwawa District, Southern Malawi, a rural area with holo-endemic malaria transmission at the time of the study, and was located in two hospitals: Chikwawa District Hospital (CDH), a government hospital with free services, and Montfort Hospital (MH) 30 km away, which is a fee-paying mission hospital.

### Study sample

All women attending the antenatal facilities of CDH or MH were screened at their first antenatal visit. Verbal informed consent was obtained. Women were encouraged to deliver in the hospital facilities and those who attended for delivery were screened a second time. Most women received two intermittent treatment doses of sulphadoxine-pyrimethamine (sulphadoxine 1,500 mg; pyrimethamine 75 mg) (IPTp-SP), one at the time of their first antenatal visit (if after the first trimester), and a second dose at between 28–34 weeks gestation [13].

### Enrolment and delivery procedures

At enrolment a questionnaire was completed on obstetric history. Mid upper-arm circumference (MUAC) was measured in women at the mid-point of the left arm (mm) and height (cms) using a height board. Each subject was given a month’s supply of haematinics (ie, 30 tablets containing 200 mg ferrous sulphate and 250 μg folic acid) at every antenatal visit and asked to visit monthly. At-delivery frequency of use of sulphadoxine-pyrimethamine was available from the antenatal card. A venipuncture blood sample was collected in an EDTA tube at first antenatal visit and also immediately prior to delivery. Within six hours of collection, haemoglobin (Hb) concentration was measured photometrically by conversion to cyanomethemoglobin (Biotron hemoglobinometer) using a standard control. Within 24 hours packed cell volume (PCV) was measured by micro-haematocrit), and ZPP in μg ZP/gHb (AVIV hematofluorometer). ZPP measurements were standardised against manufacturer control samples. Mean corpuscular haemoglobin concentration (MCHC) was calculated from Hb and PCV. Sera of mothers, whose babies were included in a separate infant follow-up study, were HIV tested after maternal informed consent and counselling. Maternal HIV infection was defined by presence of HIV antibodies determined by ICE* HIV-1.02 (Minex, Dartford, UK) and confirmed by VIDAS HIV-2 test (Bio Merieux, Lyon, France). At the time of the study, antiretroviral therapy was not available, nor Co-trimoxazole prophylaxis, so women did not receive these drugs. Malaria slides were prepared and stained with Giemsa and read counting asexual *Plasmodium falciparum* parasites against 200 white blood cells. A malaria smear was prepared from blood obtained from 1 cm placental villous incision.

### Definitions

Anaemia was defined as Hb <11 g/dl, severe anaemia as Hb <8 g/dl, and iron-deficient erythropoiesis as ZPP concentration > 2.7 μg ZP/g Hb [14,16]. Low MCHC was defined as <32 g/d [17]. Longitudinal iron transition categories (iron deficient or replete) were defined, based on raised or normal ZPP categories, for women screened at both first antenatal visit and at delivery. The four longitudinal transition categories refer to subjects with ZPP and malaria data both at first antenatal visit and at delivery. These were: (1) iron replete at first antenatal visit and at delivery; (2) iron deficient at first antenatal visit and iron replete at delivery; (3) iron deficient at first antenatal visit and at delivery; (4) iron replete at first antenatal visit and iron deficient at delivery. Peripheral parasitaemia refers to detection of *P. falciparum* parasites on a blood smear from a venipuncture sample collected at first antenatal visit or delivery. Placental parasitaemia was a positive *P. falciparum* blood smear obtained from a sample collected following placental villous incision.

### Analysis

Cross-sectional analyses measured malaria parasitaemia prevalence and ZPP concentration at either first antenatal visit or at delivery. Malaria prevalence was estimated for women with normal or raised ZPP levels. Longitudinal analyses classified ZPP transition categories (raised or normal values), which occurred between the first antenatal visit and delivery, and related these to either placental or peripheral parasitaemia prevalence at delivery. Data were analysed using SPSS for Windows version 18. Discrete variables were compared using chi-square tests. For the longitudinal data set multivariate logistic regression was used to calculate odds ratios for factors associated with delivery malaria as the dependent variable (peripheral or placental parasitaemia). Factors included in the regression models were intermittent use of sulphadoxine-pyrimethamine in pregnancy (IPTp), gravidity, maternal HIV status, and longitudinal ZPP transition category.

Ethical approval for the study was granted by the Malawi Health Science and Research Committee, and the Liverpool School of Tropical Medicine.

## Results

**Figure 1:**
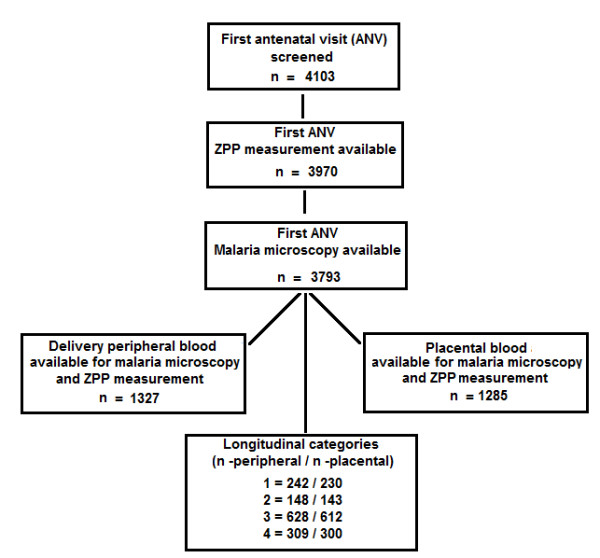
**Cross-sectional and longitudinal sample sizes.** Cross-sectional refers to samples available at either first antenatal visit or at delivery. Longitudinal categories refers to the four longitudinal transition categories of subjects with ZPP and malaria data both at first antenatal visit and at delivery. These were: (1) iron replete at first antenatal visit and at delivery; (2) iron deficient at first antenatal visit and iron replete at delivery; (3) iron deficient at first antenatal visit and at delivery; (4) iron replete at first antenatal visit and iron deficient at delivery.

The sample sizes available for the analysis are shown in Figure 1. At the first antenatal visit (n = 4,103) blood clotting or haemolysis prevented determination of ZPP for 133 women (3.2 %). Of women with a ZPP measurement 3793 (95.5 %) had an available malaria smear. The number of placental blood samples was lower than for peripheral blood at delivery as a small number of placentas were not available for sampling (placental retention, unsuitable specimen or discarded before sampling). HIV status was available for 630 women.

Prevalence of demographic and nutritional characteristics at first antenatal visit is shown in Table 1. Mean gestational age at first attendance for primigravidae was 20.5 weeks (SD ± 2.8), and multigravidae 21.9 weeks (± 6.2). One fifth of subjects were adolescent (21.7 %), or primigravidae (19.9 %). Most women were anaemic and those with parasitaemia were mainly asymptomatic. Approximately a quarter of primigravidae and two thirds of multigravidae were iron deficient based on the ZPP measurement alone. Malaria prevalence was significantly higher in primigravidae than multigravidae (P < 0.001). HIV infection prevalence was 25.4 %.

**Table 1:** Participant characteristics at first antenatal visit

**Characteristic**			**n**	**%**
Age ≤19 years*			2,839	21.7
Haemoglobinconcentration atfirst antenatal visit (1^st^ ANV) Hb < 11 (g/dL)			4,071	88.9
Mid- upper arm circumference ( <23 cm)			3,746	7.4
Primigravidae			4,103	19.9
Secundigravidae			4,103	17.3
Multigravidae			4,103	62.8
Body mass index (BMI) (<18.5 kg/m^2)^			4,037	7.5
Nutrition status (BMI < 18.5 and MUAC <23 cm)			4,002	2.6
ZPP >2.7 μg ZP/g Hb	1^st^ ANV	All gravidae	3,970	44.9
		Primigravidae	788	23.1
		Secundigravidae	690	17.8
		Multigravidae	2,492	59.1
MCHC <32.0 g/dL	1^st^ ANV	All gravidae	3,729	45.6
		Primigravidae	752	20.9
		Secundigravidae	650	17.8
		Multigravidae	2,327	61.4
Peripheral malaria at 1^st^ANV		Primigravidae	779	35.3
		Secundigravidae	673	21.2
		Multigravidae	2,462	13.2
HIV positive		All gravidae	630	25.4
		Primigravidae	158	19.6
		Multigravidae	472	27.3

n: available sample.

*For participants who knew their age.

BMI: body mass index: weight, kg/ height, m^2^ ; ANV : antenatal visit.

### Cross-sectional analysis

Table 2 summarizes malaria prevalence at first antenatal visit and at delivery, stratified by raised and/or normal ZPP values. At first antenatal visit, and for all gravidae classes, malaria prevalence was higher in women with raised ZPP values (ZPP >2.7 μgZP/gHb) (p = 0.048). At delivery there was also a higher peripheral parasitaemia prevalence with raised ZPP values for all gravidae combined (p < 0.001). Placental malaria prevalence was higher for all gravidae in women with raised ZPP values, although this difference was not significant.

**Table 2:** Malaria prevalence at first antenatal visit and delivery by ZPP concentration

**Gravida**	**ZPP, μg ZP/g Hb**		**RR (95 % CI) P value**
	**>2.7**	**≤2.7**	
First ANV	% (n)	% (n)	
All Gravida	20.0 (2218)	17.5 (1575)	1.15 (1.00 – 1.31) 0.048
PG	36.3 (513)	32.6 (242)	1.11 (0.90 – 1.38) 0.33
SG	20.6 (393)	22.5 (262)	0.92 (0.68 – 1.23) 0.56
MG	13.5 (1311)	12.8 (1071)	1.06 (0.86 – 1.30) 0.61
Delivery			
All Gravida	23.8 (960)	15.2 (407)	1.56 (1.21 – 2.02) <0.001
PG	38.7 (217)	26.8 (82)	1.44 (0.97 – 2.14) 0.056
SG	26.4 (163)	20.6 (63)	1.28 (0.74 – 2.21) 0.37
MG	17.4 (580)	10.3 (262)	1.69 (1.13 – 2.52) 0.008
Placenta			
All Gravida	19.0 (935)	15.6 (390)	1.22 (0.93 – 1.59) 0.14
PG	34.6 (211)	31.3 (80)	1.11 (0.76 – 1.61) 0.59
SG	21.8 (156)	18.2 (55)	1.20 (0.64 – 2.26) 0.57
MG	12.5 (568)	10.2 (255)	1.23 (0.80 – 1.87) 0.34

Brackets: available sample.

ZPP: zinc erythrocyte protoporphyrin.

ANV: antenatal visit.

PG: primigravida; SG: secundigravida; MG: multigravida; RR: relative risk.

There were no differences in malaria prevalence at either first antenatal visit or delivery (peripheral or placental) between women with normal or low MCHC values, except for multigravidae at first antenatal visit with low MCHC, who had lower malaria prevalence, compared to those with normal MCHC (12.1 % versus 15.1 %, 0.80, 0.64-1.00, p = 0.048).

### Longitudinal analysis

Prevalence estimates for peripheral or placental malaria at delivery for the four iron transition categories based on ZPP measurements are shown in Table 3. Delivery malaria prevalence was lowest (peripheral or placental blood) in women who were classified as iron deficient at first antenatal visit and replete at delivery (category 2) when compared with other ZPP transition categories. This difference was significant for peripheral parasitaemia in primigravidae (relative risk 0.34, 95 % CI 0.14-0.83). Women with raised ZPP values at first antenatal visit and at delivery (possible chronic iron deficiency; category 3) had higher malaria prevalence at delivery than women with normal ZPP values at both the antenatal visit and at delivery (possible iron replete), (all gravidae: 1.38, 1.01-1.87; multigravidae: 1.52, 1.01-2.27).

**Table 3:** ***Plasmodium falciparum*prevalence (%) and risk estimates at delivery in four ZPP transition categories by gravida**

**Iron Transition**	**Peripheral parasitaemia**			**Placental parasitaemia**		
	**All Gravida**	**PG**	**MG**	**All Gravida**	**PG**	**MG**
	**RR(95%CI)**	**RR(95%CI)**	**RR(95%CI)**	**RR(95%CI)**	**RR(95%CI)**	**RR(95%CI)**
ZPP						
Category	17.4 (242)	39.0 (41)	12.9 (201)	18.3 (230)	40.0 (40)	13.7 (190)
1	Refs	Refs	Refs	Refs	Refs	Refs
Category	11.5 (148)	13.2 (38)	10.9 (110)	11.9 (143)	21.6 (37)	8.5 (106)
2	0.7	0.3	0.8	0.6	0.5	0.6
	(0.4-1.1)	(0.1-0.8)	(0.4-1.6)	(0.4-1.1)	(0.3-1.1)	(0.3-1.3)
Category	23.9 (628)	37.0 (154)	19.6 (474)	20.4 (612)	34.5 (148)	15.9 (464)
3	1.4	0.9	1.5	1.1	0.9	1.2
	(1.0-1.9)	(0.6-1.5)	(1.0-2.3)	(0.8-1.5)	(0.6-1.3)	(0.8-1.8)
Category	23.0 (309)	44.1 (59)	18.0 (250)	16.0 (300)	33.9 (59)	11.6 (241)
4	1.3	1.1	1.4	0.9	0.8	0.8
	(0.9-1.9)	(0.7-1.8)	(0.9-2.2)	(0.6-1.3)	(0.5-1.4)	(0.5-1.4)

Brackets: available sample, or 95 % confidence interval.

ZPP: zinc erythrocyte protoporphyrin;

Category 1: ZPP normal to ZPP normal.

Category 2: Raised ZPP to normal ZPP.

Category 3: Raised ZPP to raised ZPP.

Category 4: Normal ZPP to raised ZPP.

Table 4 summarizes participant characteristics for the four ZPP transition categories. IPTp uptake was highest in women in category 2 (p < 0.05 for all category comparisons). Other characteristics did not differ significantly between categories, except for anaemia (Hb <11 g/dl and <8 g/dl), which had lowest prevalence in category 1 (iron replete at both visits) (p < 0.001).

**Table 4:** Prevalence (%) of study characteristics in the four longitudinal ZPP transition categories

**Characteristic**	**ZPP Category**			
	**1**	**2**	**3**	**4**
Primigravidae	17.3 (249)	25.5 (157)	24.6 (642)	18.8 (320)
IPTp-SP ≥1dose	72.0 (246)	81.3 (150)	68.1 (627)	62.9 (310)
Hb < 11 g/dl	58.4 (243)	62.7 (150)	87.4 (628)	76.1 (305)
Hb < 8 g/dl	2.1 (243)	8.7 (150)	18.0 (628)	9.2 (305)
LBW < 2500 g	13.5 (245)	19.1 (152)	19.0 (633)	15.2 (309)
HIV positive	23.7 (97)	37.7 (61)	20.5 (268)	30.0 (130)

Brackets: available sample.

IPTp-SP: intermittent preventive treatment with sulfadoxine – pyrimethamine;

Hb:hemoglobin; LBW: low birth weight; ZPP: zinc erythrocyte protoporphyrin.

Category 1: ZPP normal to ZPP normal.

Category 2: Raised ZPP to normal ZPP.

Category 3: Raised ZPP to raised ZPP.

Category 4: Normal ZPP to raised ZPP.

For the longitudinal data set logistic regression analysis using two models was performed with malaria at delivery as the dependent variable. Models were based on the ZPP cut-off used to define iron deficiency. Factors included were gravidae, IPTp uptake ≥ one dose, and ZPP transition categories 1–4. The second model included HIV status as only a subsample of women (16 %) had known HIV status. Risk of malaria at delivery (peripheral or placental) was higher in primigravidae (p < 0.001), and lower (peripheral) with IPTp use (p < 0.001). HIV infection was associated with increased malaria risk (p < 0.02, peripheral or placental). There was lower risk of peripheral parasitaemia at delivery for women who transitioned from raised ZPP at first antenatal visit to normal ZPP values at delivery (p = 0.048) (ZPP category 2). This reduced risk remained in model 2, which included HIV status, but was no longer significant.

## Discussion

This is the first analysis of ZPP as a potential iron biomarker of malaria risk in pregnancy. Use of ZPP transition categories allowed malaria risk to be assessed concurrently with longitudinal changes in this iron biomarker. Gestational iron status is influenced by antenatal haematinic use, iron bioavailability [18] and enteric iron losses. Under the high malaria exposure in which these women were living, a higher malaria prevalence in primigravidae than multigravidae would be expected [19], and was observed across all ZPP transition categories, indicating the substantial influence of parity on malaria susceptibility under these malaria-endemic conditions. Almost two thirds of multigravidae and a quarter of primigravidae would be classified as iron deficient, based on either the ZPP measurement alone, and approximately 90 % were anaemic.

ZPP reflects bone marrow iron requirements [20], and as ZPP and haemoglobin are diluted equally during pregnancy due to plasma volume expansion, the use of a ZPP/haemoglobin index avoids misinterpretation that may result from plasma volume changes. In a non-malarious area the diagnostic sensitivity and predictive value of ZPP for evaluating iron depletion and risk of anaemia in pregnancy compared favourably to those of ferritin and transferrin saturation measurements.[21]. Expansion of plasma volume is greater than that of red cell mass and leads to a decline in haemoglobin and haematocrit [18], with little change in average red-cell haemoglobin concentration, or MCHC [16]. MCHC is, therefore, a useful indicator of iron deficiency although by definition it can only diagnose iron deficiency at the stage of anaemia. Based on a MCHC <32 g/dl, approximately one in five primigravidae and three in five multigravidae would be classified as iron deficient. MCHC may be a poor biomarker when calculated from microhaematocrit without frequent calibration.

In the cross-sectional analysis at first antenatal visit, malaria prevalence was higher in women with raised ZPP values for all gravidae classes except secundigravidae. At delivery, malaria prevalence (peripheral or placental blood) was also higher in woman with raised ZPP concentrations. This association in the cross-sectional study of higher malaria prevalence in women with elevated ZPP levels is the statistically most significant finding as in the longitudinal sub-group analyses sample sizes are much smaller. The association may result from inflammation increasing ZPP concentration, rather than indicating an association of increased malaria risk with maternal iron deficiency. This is because, based on MCHC, multigravidae at first antenatal visit who had low MCHC values also had lower malaria prevalence compared to those with normal MCHC, suggesting that iron deficiency may reduce malaria risk. This suggests ZPP is not a good biomarker for iron status in this population. A limitation of ZPP as an indicator of pregnancy iron status relates to factors known to elevate its concentration, including infection [22], impaired iron utilization in chronic inflammation [23], chronic malarial haemolysis sufficient to result in iron demand in excess of the iron transport system’s ability to deliver iron to the red cell, and drug intake, for example with cotrimoxazole [20]. All of these factors are common in women living under conditions of high malaria exposure and some of these factors may explain the association in the present study of raised ZPP values with increased malaria prevalence. Raised bilirubin concentration, secondary to haemolysis could also interfere with haematoflourometer readings when whole blood samples were used [24,25], and use of washed red cells would have been preferable. Very high levels of zinc protoporphyrin may be toxic to the malaria parasite [26], and in very anaemic Tanzanian children reduced malaria risk was reported with raised ZPP concentration [11].

In the present study gravidae who maintained normal ZPP concentrations at first antenatal visit and at delivery (i e, possibly iron replete throughout pregnancy), had higher delivery malaria prevalence than those who transitioned from raised to normal ZPP values (i e, became iron replete in late pregnancy). This might suggest that continuous iron repletion during gestation was associated with higher delivery malaria risk, and not late gestational iron repletion. But women with continuous iron repletion did not have higher delivery malaria prevalence than women who transitioned to raised ZPP values at delivery (i e, were iron deficient late in pregnancy, or throughout pregnancy), suggesting that if ZPP is accurately indicating maternal iron status then it is not discriminating malaria risk, as both chronically iron-replete and chronically iron-deficient women were at equivalent risk of malaria at delivery. However, if malaria-related inflammation raises ZPP concentration, independent of maternal iron status, then gestational transition to lower ZPP values would be associated with a reduction in malaria prevalence at delivery compared to women who show no change in ZPP concentration, as shown in this study.

In regression analysis, peripheral or placental malaria was positively associated with primigravidae and HIV infection, and lower malaria risk was associated with uptake of one or more doses of IPTp-SP. These associations would be expected and their magnitude provides a useful gauge against which to assess malaria risk estimates related to ZPP categories. Transition to normal ZPP concentration was associated with reduced risk for malaria parasitaemia in peripheral or placental blood (Table 5). These findings are consistent with data from the cross-sectional analysis at first antenatal visit and delivery and suggest that malaria parasitaemia elevates ZPP concentration. If antiretroviral therapy were available, as was not the case at the time of this study, then the host inflammatory response may be less marked, and as a consequence ZPP as a biomarker of iron status might have better validity.

**Table 5:** Multivariate analysis of malaria at delivery

**Factors**	**Peripheral parasitaemia**	**Placental parasitaemia**
	**AOR (95 % CI) P value**	**AOR (95 % CI) P value**
**Model 1 ZPP**		
Primigravidae	2.71 (2.02 – 3.65) 0.001	3.18 (2.34 – 4.33) 0.001
IPTp-SP ≥1dose	0.60 (0.44 – 0.82) 0.001	0.91 (0.66 – 1.26) 0.56
ZPP Category 1	0.68 (0.44 – 1.05) 0.08	1.22 (0.77 -1.94) 0.40
ZPP Category 2	0.54 (0.29 – 0.99) 0.05	0.54 (0.29 – 1.00) 0.05
ZPP Category 3	1.46 (0.99 – 2.15) 0.06	1.06 (0.71 – 1.58) 0.79
ZPP Category 4	1.48 (0.96 – 2.29) 0.08	0.83 (0.52 – 1.32) 0.43
**Model 2 ZPP and HIV Status**		
Primigravidae	2.44 (1.59 – 3.73) 0.001	2.84 (1.82 – 4.41) 0.001
HIV positive	1.67 (1.07 – 2.62) 0.03	1.75 (1.09 – 2.80) 0.02
IPTp-SP ≥1dose	0.65 (0.42 – 1.02) 0.06	0.93 (0.59 – 1.47) 0.76
ZPP Category 1	0.59 (0.31 – 1.11) 0.10	1.20 (0.61 – 2.37) 0.60
ZPP Category 2	0.68 (0.29 – 1.60) 0.37	0.67 (0.28 – 1.58) 0.36
ZPP Category 3	1.42 (0.80 – 2.54) 0.23	1.16 (0.66 – 2.07) 0.63
ZPP Category 4	1.71 (0.90 – 3.24) 0.10	0.83 (0.42 – 1.65) 0.60

IPTp-SP: intermittent preventive treatment with sulphadoxine–pyrimethamine.

ZPP: Zinc erythrocyte protoporphyrin.

Category 1: ZPP normal to ZPP normal.

Category 2: Raised ZPP to normal ZPP.

Category 3: Raised ZPP to raised ZPP.

Category 4: Normal ZPP to raised ZPP.

A proportion of women in this population will have raised ZPP concentrations solely related to maternal iron deficiency and not related to inflammation. A limitation of this analysis is that without additional sensitive serum biomarkers of iron status it is not possible to discriminate this group from those with raised ZPP values due to inflammation alone. For this reason it is not possible to determine from ZPP measurements alone whether iron deficiency or repletion alters malaria susceptibility in these pregnant women. The proportions of women with raised ZPP values due to iron deficiency, or due to inflammation will also vary between gravidae as iron deficiency is generally more frequent in multigravidae [27].

## Conclusion

Analysis of ZPP as an iron biomarker from this large pregnancy data set provides evidence that elevation of ZPP concentration was associated with increased risk of malaria infection, and that this association was secondary to inflammation rather than indicating increased malaria risk with maternal iron deficiency. It was not possible from ZPP measurements alone to determine whether iron deficiency or repletion alters malaria susceptibility in pregnancy.

## Abbreviation

ZPP, Zinc erythrocyte Protoporphyrin.

## Competing interests

The author(s) declare that they have no competing interests'.

## Author’s contributions

ES and GK analysed the data and assisted in writing the manuscript. BJB was principal investigator for the field study and contributed to data analysis and writing the paper. All authors read and approved the final manuscript.
